# Visceral adiposity is associated with altered myocardial glucose uptake measured by ^18^FDG-PET in 346 subjects with normal glucose tolerance, prediabetes, and type 2 diabetes

**DOI:** 10.1186/s12933-015-0310-4

**Published:** 2015-11-04

**Authors:** Gyuri Kim, Kwanhyeong Jo, Kwang Joon Kim, Yong-ho Lee, Eugene Han, Hye-jin Yoon, Hye Jin Wang, Eun Seok Kang, Mijin Yun

**Affiliations:** Division of Endocrinology and Metabolism, Department of Internal Medicine, Yonsei University College of Medicine, 50-1, Yonsei-ro, Seodaemun-gu, Seoul, 03722 Republic of Korea; Graduate School, Yonsei University College of Medicine, Seoul, 03722 Republic of Korea; Department of Nuclear Medicine, Yonsei University College of Medicine, 50-1, Yonsei-ro, Seodaemun-gu, Seoul, 03722 Republic of Korea; Brain Korea 21 PLUS Project for Medical Science, Yonsei University College of Medicine, Seoul, 03722 Republic of Korea; Institute of Endocrine Research, Yonsei University College of Medicine, Seoul, 03722 Republic of Korea

**Keywords:** Visceral fat, Myocardium, Type 2 diabetes mellitus, Positron emission tomography, Insulin resistance

## Abstract

**Background:**

The heart requires constant sources of energy mostly from free fatty acids (FFA) and glucose. The alteration in myocardial substrate metabolism occurs in the heart of diabetic patients, but its specific association with other metabolic variables remains unclear. We aimed to evaluate glucose uptake in hearts of subjects with normal glucose tolerance (NGT), prediabetes, and type 2 diabetes mellitus (T2DM) using [^18^F]-fluorodeoxyglucose-positron emission tomography (^18^FDG-PET) in association with visceral and subcutaneous adiposity, and metabolic laboratory parameters.

**Methods:**

A total of 346 individuals (NGT, n = 76; prediabetes, n = 208; T2DM, n = 62) in a health promotion center of a tertiary hospital were enrolled. The fasting myocardial glucose uptake, and visceral and subcutaneous fat areas were evaluated using ^18^FDG-PET and abdominal computed tomography, respectively.

**Results:**

Myocardial glucose uptake was significantly decreased in subjects with T2DM compared to the NGT or prediabetes groups (p for trend = 0.001). Multivariate linear regression analyses revealed that visceral fat area (β = −0.22, p = 0.018), fasting FFA (β = −0.39, p < 0.001), and uric acid levels (β = −0.21, p = 0.007) were independent determinants of myocardial glucose uptake. Multiple logistic analyses demonstrated that decreased myocardial glucose uptake (OR 2.32; 95 % CI 1.02–5.29, p = 0.045) and visceral fat area (OR 1.02, 95 % CI 1.01–1.03, p = 0.018) were associated with T2DM.

**Conclusions:**

Our findings indicate visceral adiposity is strongly associated with the alteration of myocardial glucose uptake evaluated by ^18^FDG-PET, and its association further relates to T2DM.

## Background

Type 2 diabetes mellitus (T2DM) is a chronic and increasing worldwide disorder that is characterized by hyperglycemia, insulin resistance, and dyslipidemia, and its independent association with dysfunctional adiposity, such as excess visceral fat has been reported [1, 2]. Several studies demonstrated that T2DM also provokes long-term dysfunction and damage to various organs including the eye, kidney, and heart [3]. In general, the heart requires constant sources of energy that mainly include free fatty acids (FFA) and glucose for continuous pumping, and has high flexibility with regards to the energy substrate metabolism [4]. However, in the T2DM heart, alterations in myocardial substrate metabolism, characterized by increased myocardial fatty acid metabolism and concurrently decreased glucose metabolism, have been reported [4–6].

Positron emission tomography (PET) can monitor increased uptake of the glucose analogue [^18^F]-fluorodeoxyglucose (^18^FDG), that is taken up by tissues via glucose transporter proteins [7]. Recent studies have reported that disturbances of carbohydrate, fat, and protein metabolism altered biodistribution of ^18^FDG in patients with T2DM [8]. In addition, regarding myocardial insulin sensitivity, myocardial glucose uptake can differ according to the status of whole body insulin resistance, such as prediabetes and T2DM, compared with normal glucose tolerance (NGT) controls. However, few studies have investigated the relationship(s) of myocardial glucose uptake using PET, adiposity, and other metabolic profiles in subjects with NGT, prediabetes, and T2DM [8–10].

In the current study, we therefore investigated myocardial glucose uptake using [^18^F]-fluorodeoxyglucose-positron emission tomography (^18^FDG-PET), and characterized its associations with various clinical and laboratory parameters according to glycemic status.

## Methods

### Study population

Between January, 2008 and July, 2014, asymptomatic 387 individuals who visited the health promotion center in Severance Hospital for comprehensive health examinations were included on this study. Patients with present abnormal renal or hepatic functions, history of myocardial infarction, and heart failure were excluded (n = 20). Furthermore, 23 subjects with coronary artery calcium score (CACS) over 400 evaluated by coronary computed tomography angiography were excluded for subclinical coronary artery disease [11]. Finally, we studied a total of 346 individuals including 76 healthy subjects with NGT, 208 subjects with prediabetes such as impaired fasting tolerance, impaired glucose tolerance, and 62 subjects with T2DM, as defined by the 2014 revision of the American Diabetes Association guidelines [12]. The protocol of this study adhered to the tenets of the Declaration of Helsinki, was approved by the Institutional Review Board (IRB No. 4-2015-0038) of Severance Hospital.

### Measurement of clinical and laboratory parameters

All individuals provided data including personal medical history, drinking and smoking habits, and use of medication at the time of their enrolment. Drinking habits were categorized according to self-reports as noncurrent or current, and smoking habits as never, ex-smoker, and current smoker. Body mass index (BMI) was defined as weight divided by the square of the height (kg/m^2^). Blood pressure was obtained by averaging the results of three blood pressure recordings in the sitting position, each after at least 5 min of rest. Blood samples were collected from each participant after overnight fasting. The fasting and 2 h postprandial glucose, insulin, FFA, and triglycerides (TG) were measured. Plasma glucose was mea-sured using the glucose oxidase method. Plasma total choles-terol, TG, high density lipoprotein (HDL) cholesterol, FFA, lipoprotein(a), and uric acid were assayed us-ing a Hitachi 7600 Auto Analyzer (Hit-achi Instruments Service, Tokyo, Japan). Low density lipopro-tein (LDL) cholesterol was calculated using the Friedewald equation (LDL cholesterol [mg/dL] = total cholesterol [mg/dL] − HDL cholesterol [mg/dL] − TG [mg/dL]/5). Serum glycated albumin was determined using an enzymatic method as previously described [13]. HbA_1c_ was measured by high-performance liquid chromatography using a Variant™ II Turbo (Bio-Rad Laboratories, Hercules, CA, USA). Homeostasis Model Assessment of Insulin Resistance (HOMA-IR) was calculated using the following formula: (fasting plasma insulin [μU/mL] × fasting plasma glucose [mg/dL]/405) [14]. An index of Adipose tissue Insulin Resistance (Adipo-IR) was calculated using the following formula: (fasting plasma FFA [mmol/L] × fasting plasma insulin [pmol/L]) [15].

### Abdominal adipose tissue areas and coronary artery calcification score (CACS) by multislice computed tomography (CT)

The abdominal adipose tissue areas were determined by either a dual source 128 slice CT scanner (Somatom Definition Flash, Siemens Healthcare, Forchheim, Germany) or a 64 slice CT scanner (Somatom Sensation 64, Siemens Healthcare) with a slice thickness of 3 mm, a tube voltage of 120 kV, 150 effective mAs, 1.0 pitch, and a 0.5 s rotation time. Visceral adipose tissue, and subcutaneous adipose tissue areas at the L3–L4s vertebral disc space in a supine position were measured using the Aquarius iNtuition Viewer software, version 4.4.11 (Terarecon, San Mateo, CA, USA). The fat area was identified using attenuation values between −190 and −30 Hounsfield units. For CACS, a prospective electrocardiography-gated scan was performed with a slice-width of 3 mm, a tube voltage of 100 kV, 80 mAs, collimation 32 × 1.2, and table feed of 34.5 mm. The images were reconstructed with the B36f kernel (b36f), and CACS was calculated with the use of CAC analysis software (Cascore, Siemens, Germany) using the Agatstone method [16].

## ^18^FDG-PET and image analysis

Whole body PET-CT was performed using either one of two combined PET-CT scanners: a Biograph TruePoint 40 (Siemens Medical Solutions, Hoffman Estates, IL, USA) or a Discovery 600 (General Electric Medical Systems, Milwaukee, WI, USA). All patients fasted for at least 8 h, and blood glucose levels were recorded before the injection of ^18^FDG. Approximately 5.5 mBq of ^18^FDG per kilogram of body weight were administered intravenously. PET-CT scanning was conducted from the skull base to the mid-thigh at 60 min after injection. For the Biograph TruePoint 40 scanner, a spiral CT scan with a 0.5 s rotation time, 35 mA, 120 kVp, and 5 mm section width with arms raised, was used. For the Discovery 600 scanner, a spiral CT scan with a 0.8 s rotation time, 60 mA, 120 kVp, 3.75 mm section thickness, 1.25 mm collimation, and 27.5 mm table feed per rotation with arms raised, was used. PET image acquisition followed CT scanning using the following parameters: 2.5 min per bed position of 21.6 cm in a three-dimensional acquisition mode (Biograph TruePoint 40) or 2 min per bed position of 15.7 cm in a three-dimensional acquisition mode (Discovery 600). Reconstructions of PET images were acquired using a 128 × 128 matrix with ordered subset expectation maximization and attenuation correction.

The standardized uptake value (SUV) was calculated by nuclear medicine experts who were blind to the subjects’ clinical and laboratory data as follows: SUV = (decay-corrected activity [kBq] per mL of tissue volume)/(injected ^18^FDG activity [kBq]/body mass [g]). Two-dimensional regions of interest (ROIs) were drawn through the transaxial images to measure the SUV_max_ of the left ventricular myocardium. Patients with striking focal FDG uptake in the left ventricle that could be caused by ischemic change were excluded [17]. We also obtained the liver SUV, which was quite stable over time, from the circular ROI along the periphery of the right lobe, 1 cm from the margin by averaging at least three times of these values. The values of SUV of the heart to liver FDG uptake ratio (SUV_Heart_/SUV_Liver_) were used to estimate the myocardial glucose uptake to minimize variability [18–20].

### Statistical analysis

All continuous variables were expressed as the mean ± standard deviation, and categorical variables were expressed as proportions. Differences were analysed using the analysis of variance (ANOVA) for continuous variables and the Chi-square test for categorical variables. Comparisons of myocardial glucose uptake relative to the status of diabetes were calculated with the Jonckheere–Terpstra trend test. Pearson’s correlation coefficients were calculated to examine the relationships between myocardial glucose uptake and metabolic variables. Multiple linear regression analysis was performed to determine the independent relationships of the studied variables, and standardized β was represented as the coefficient β. The odds ratios (ORs) and 95 % confidence intervals (CIs) for the factors associated with T2DM were calculated using the multiple logistic regression analysis. In the Pearson’s correlation, multiple linear regression, and multiple logistic regression analysis, values of myocardial glucose uptake (SUV_Heart/Liver_) were log-transformed to achieve normal distribution. A value of p < 0.05 was considered statistically significant. Statistical analyses were performed using PASW Statistics software, version 20.0 for Windows (SPSS Inc., Chicago, IL, USA).

## Results

### Baseline characteristics of the study population

For all subjects, the mean age of patients was 56.9 ± 10.7 years and 53.1 % were women. The average BMI was 24.1 ± 3.4 kg/m^2^, which was within the overweight range of BMI (23.0–24.9 kg/m^2^) by Asia–Pacific BMI cutoffs [21]. Of these 346 subjects, the total number of patients who were taking one or more antihypertensive medications were 75 (22.0 %) and lipid lowering medications were 63 (18.4 %; statin, n = 57, 16.5 %; fenofibrate, n = 4, 1.2 %; omega-3, n = 4, 1.2 %). Among T2DM patients, oral antidiabetic drug users and insulin users were 32.8 and 8.2 %, respectively. The baseline characteristics of participants by glycemic status are shown in Table 1. Subjects with prediabetes or T2DM tended to be older, to have higher systolic blood pressure, to be more obese, and to have metabolically unhealthy factors compared to subjects with NGT. Subjects with prediabetes or T2DM were more likely to have higher levels of uric acid and larger visceral fat areas compared to subjects with NGT.

**Table 1:** Characteristics of the study subjects

Variables	NGT (*n* = 76)	Prediabetes (*n* = 208)	T2DM (*n* = 62)	p
Age (years)	50.1 ± 10.9	57.9 ± 9.1	61.7 ± 11.5	<0.001
Female sex	38 (50.0)	101 (48.6)	37.1 (62.9)	0.233
Current drinker	30 (40.5)	110 (53.9)	27 (44.3)	0.099
Current smoker	11 (14.9)	27 (13.2)	8 (13.1)	0.934
SBP (mmHg)	117.7 ± 13.3	121.5 ± 14.3	127.9 ± 12.9	<0.001
DBP (mmHg)	74.9 ± 10.8	77.0 ± 10.1	79.7 ± 10.5	0.036
BMI (kg/m^2^)	22.9 ± 2.9	24.1 ± 3.1	25.6 ± 4.0	<0.001
Visceral fat area (cm^2^)	104.1 ± 46.8	126.1 ± 59.4	176.7 ± 77.3	<0.001
Subcutaneous fat area (cm^2^)	132.6 ± 40.8	143.7 ± 55.9	161.9 ± 76.0	0.064
HbA_1c_ (*%*)	5.5 ± 0.2	5.9 ± 0.2	6.8 ± 1.1	<0.001
HbA_1c_ (mmol/mol)	36.6 ± 2.1	41.0 ± 2.1	50.8 ± 11.6	<0.001
Glycated albumin (%)	11.0 ± 1.3	11.6 ± 1.5	15.5 ± 5.7	<0.001
Fasting glucose (mg/dL)	90.0 ± 8.1	96.6 ± 9.4	115.4 ± 28.5	<0.001
Postprandial glucose (mg/dL)	100.5 ± 14.1	118.4 ± 31.4	27.1 ± 58.6	<0.001
Fasting insulin (µIU/mL)	5.46 ± 3.18	7.06 ± 4.14	8.62 ± 7.14	0.002
Postprandial insulin (µIU/mL)	21.61 ± 16.55	35.13 ± 31.95	54.85 ± 53.23	<0.001
Fasting C-peptide (ng/mL)	1.69 ± 0.61	2.15 ± 0.75	2.36 ± 1.12	<0.001
Postprandial C-peptide (ng/mL)	5.29 ± 2.61	7.18 ± 3.54	7.81 ± 4.67	<0.001
HOMA-IR	1.21 ± 0.73	1.70 ± 1.06	2.65 ± 2.40	<0.001
TyG	8.25 ± 0.57	8.50 ± 0.50	8.80 ± 0.63	<0.001
Adipo-IR	25.93 ± 20.59	30.89 ± 23.76	37.53 ± 37.47	0.284
Total cholesterol (mg/dL)	191.4 ± 36.8	191.5 ± 38.1	166.6 ± 45.3	0.001
HDL cholesterol (mg/dL)	49.1 ± 11.8	49.9 ± 12.6	45.0 ± 11.7	0.018
LDL cholesterol (mg/dL)	114.9 ± 33.6	114.8 ± 33.6	96.8 ± 33.8	0.001
Fasting TG (mg/dL)	103.0 ± 71.0	116.2 ± 59.8	133.2 ± 73.2	0.055
Postprandial TG (mg/dL)	91.1 ± 71.7	107.6 ± 50.6	132.7 ± 84.4	0.138
Fasting FFA (µEq/L)	690.4 ± 380.4	621.4 ± 238.6	704.3 ± 305.4	0.298
Postprandial FFA (µEq/L)	171.5 ± 182.9	134.0 ± 104.1	191.8 ± 154.9	0.126
Lipoprotein(a) (mg/dL)	22.1 ± 24.1	23.4 ± 25.4	29.7 ± 34.2	0.359
Uric acid (mg/dL)	4.9 ± 1.3	5.5 ± 1.3	5.4 ± 1.1	<0.001
Antihypertensive medication user	10 (13.3)	42(20.5)	23 (37.7)	0.002
Statin user	6(7.9)	31(14.9)	20 (32.3)	<0.001
Antidiabetic medication user	NA	NA	25 (41.0)	
Metformin user	NA	NA	19 (31.1)	
Sulfonylurea user	NA	NA	9 (14.8)	
DPP4 inhibitor user	NA	NA	9 (13.1)	
TZD user	NA	NA	5 (8.2)	
Insulin user	NA	NA	5 (8.2)	

Data are expressed as mean ± standard deviation of the mean or n (%)

*Adipo*-*IR* index of adipose tissue insulin resistance, *BMI* body mass index, *DBP* diastolic blood pressure, *DPP4* dipeptidyl peptidase-4, *FFA* free fatty acids, *HOMA*-*IR* Homeostasis model assessment of insulin resistance, *HDL* high density lipoprotein, *IFG* impaired fasting glucose, *LDL* low density lipoprotein, *NA* not applicable, *NGT* normal glucose tolerance, *SBP* systolic blood pressure, *T2DM* type 2 diabetes, *TG* triglycerides, *TyG* the product of fasting triglycerides and glucose levels, *TZD* thiazolidinedione

### Myocardial glucose uptake for NGT, prediabetes, and T2DM subjects

**Fig. 1:**
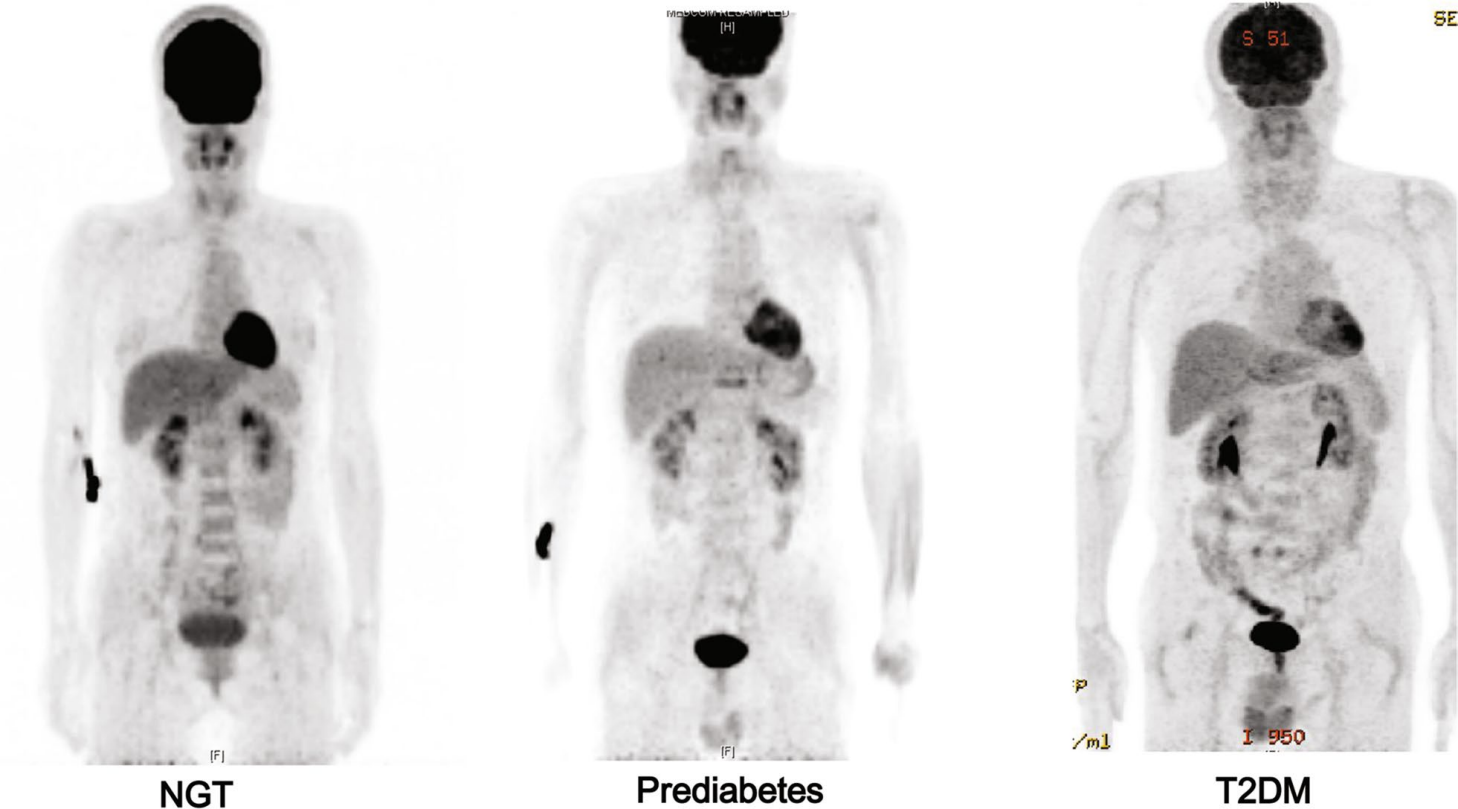
Altered myocardial glucose uptake of [^18^F]-fluorodeoxyglucose (^18^FDG) in subjects according to the glycemic status. Each subject has a median value within the highest tertile of myocardial glucose uptake for normal glucose tolerance (NGT), prediabetes, and type 2 diabetes mellitus (T2DM)

**Fig. 2:**
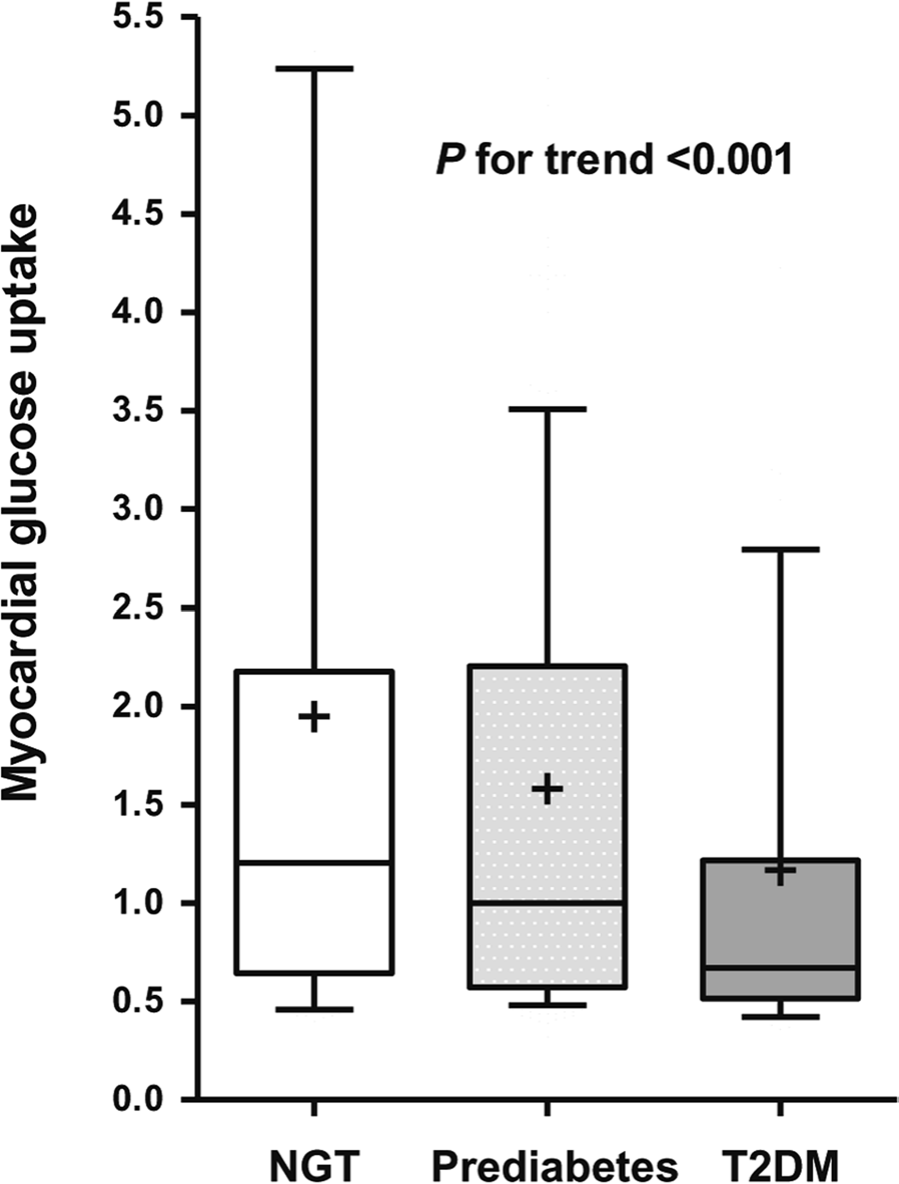
Box plot of myocardial glucose uptake according to glycemic status. The box represents the upper and lower quartiles. Each *+* and *horizontal line in the box* indicates the mean and median value of myocardial glucose uptake, respectively. The *bar* denotes 10–90 percentiles. *NGT* normal glucose tolerance, *T2DM* type 2 diabetes mellitus

Figure 1 shows the myocardial glucose uptake for subjects, based upon the glycemic status. Compared to the NGT or prediabetes groups, myocardial glucose uptake was significantly decreased in patients with T2DM (mean myocardial glucose uptake; NGT, prediabetes, and T2DM; 1.94, 1.58, and 1.17, respectively; p for trend <0.001; Fig. 2). The proportion of the highest tertile of myocardial glucose uptake was significantly lower in subjects with T2DM than those in subjects with NGT and prediabetes (p = 0.013, data not shown).

### Relationship between myocardial glucose uptake and metabolic parameters

To assess the relationship between myocardial glucose uptake and metabolic parameters, univariate analysis was performed (Table 2). Myocardial glucose uptake correlated negatively with BMI, HbA_1c_, fasting glucose, insulin, C-peptide, fasting/postprandial TG, FFA. Moreover, statistically significant inverse correlations were found between myocardial glucose uptake and evidence of insulin resistance, on the basis of HOMA-IR (r = −0.15, p = 0.011), Adipo-IR (r = −0.32, p < 0.001), uric acid (r = −0.21, p < 0.001), and visceral fat areas (r = −0.26, p < 0.001), subcutaneous fat areas (r = −0.14, p = 0.025).

**Table 2:** Correlation between myocardial glucose uptake and metabolic factors

Variables	r	p
Age (years)	−0.01	0.980
SBP (mmHg)	−0.08	0.145
DBP (mmHg)	−0.02	0.725
BMI (kg/m^2^)	−0.17	0.002
Visceral fat area (cm^2^)	−0.26	<0.001
Subcutaneous fat area (cm^2^)	−0.14	0.025
HbA_1c_ (%)	−0.12	0.027
Glycated albumin (%)	−0.01	0.896
Fasting glucose (mg/dL)	−0.16	0.002
Postprandial glucose (mg/dL)	−0.11	0.066
Fasting insulin (µIU/mL)	−0.13	0.028
Postprandial insulin (µIU/mL)	−0.11	0.069
Fasting C-peptide (ng/mL)	−0.18	0.003
Postprandial C-peptide (ng/mL)	−0.10	0.103
HOMA-IR	−0.15	0.011
TyG	−0.16	0.004
Adipo-IR	−0.32	<0.001
Total cholesterol (mg/dL)	−0.02	0.688
HDL cholesterol (mg/dL)	0.03	0.644
LDL cholesterol (mg/dL)	0.01	0.802
Fasting TG (mg/dL)	−0.16	0.003
Postprandial TG (mg/dL)	−0.23	0.003
Fasting FFA (µEq/L)	−0.33	<0.001
Postprandial FFA (µEq/L)	−0.19	0.020
Lipoprotein(a) (mg/dL)	0.05	0.378
Uric acid (mg/dL)	−0.21	<0.001

*Adipo*-*IR* index of adipose tissue insulin resistance, *BMI* body mass index, *DBP* diastolic blood pressure, *FFA* free fatty acids, *HOMA*-*IR* Homeostasis model assessment of insulin resistance, *HDL* high density lipoprotein, *LDL* low density lipoprotein, *SBP* systolic blood pressure, *TG* triglycerides, *TyG* the product of fasting triglycerides and glucose levels

Multiple linear regression analysis was performed to determine whether myocardial glucose uptake was independently associated with various factors of body composition in addition to age, sex, social habits such as drinking and smoking, use of antidiabetic medication and statin, systolic blood pressure (SBP) (Model 1), CACS and laboratory parameters in addition to the age and sex (Model 2), and variables derived from the basic models (Model 1 and 2; Model 3, Table 3). In the comprehensive model (Model 3), visceral fat area (β = −0.22, p = 0.018), fasting FFA (β = −0.39, p < 0.001), and uric acid levels (β = −0.21, p = 0.007) were independent determinants of myocardial glucose uptake after adjustment for effective parameters from Model 1 and 2.

**Table 3:** Analysis of myocardial glucose uptake associated with metabolic parameters

R^2^	Model 1		Model 2		Model 3	
	0.081		0.282		0.297	
Variables	β	p	β	p	β	p
Visceral fat area (cm^2)^	−0.29	<0.001	–	–	−0.22	0.018
Fasting FFA (µEq/L)	–	–	−0.39	<0.001	−0.39	<0.001
Uric acid (mg/dL)	–	–	−0.26	<0.001	−0.21	0.007
Postprandial glucose (mg/dL)	–	–	−0.18	0.020	–	–
Postprandial TG (mg/dL)	–	–	−0.24	0.001	–	–

*Model 1* adjusted for age, sex, current drinker, current smoker, use of antidiabetic medications, use of statin, SBP, BMI, and subcutaneous fat area, *Model 2* adjusted for age, sex, coronary artery calcification score, fasting/postprandial glucose, C-peptide, insulin, FFA, TG, HbA_1c_, uric acid, *Model 3* adjusted for age, sex, and variables derived from Model 1 and 2

*BMI* body mass index, *FFA* free fatty acids, *SBP* systolic blood pressure, *TG* triglycerides

### Associations between myocardial glucose uptake, visceral adiposity, and type 2 diabetes

As shown in Table 4, multiple logistic regression analyses were used to further investigate significant determinants in relation to T2DM. After sequential adjustment for confounding covariates including age, sex, current drinker, current smoker, use of antihypertensive medications, BMI, fasting FFA, and uric acid (Model 4), patients with T2DM had significantly elevated ORs for decreased myocardial glucose uptake (OR 2.32, 95 % CI 1.02–5.26, p = 0.045) and increased visceral fat areas (OR 1.02, 95 % CI 1.00–1.03, p = 0.018).

**Table 4:** Logistic regression analyses for related factors for type 2 diabetes

Variables	Model 1		Model 2		Model 3		Model 4	
	OR (95 % CI)	p	OR (95 % CI)	p	OR (95 % CI)	p	OR (95 % CI)	p
Myocardial glucose uptake	0.51 (0.34–0.76)	0.001	0.49 (0.31–0.77)	0.002	0.57 (0.33–0.98)	0.042	0.43 (0.19–0.98)	0.045
Visceral fat area (cm^2^)	–	–	–	–	1.01 (1.01–1.02)	0.013	1.02 (1.01–1.03)	0.018
Fasting FFA (µEq/L)	–	–	–	–	–	–	1.00 (0.99–1.00)	0.920
Uric acid (mg/dL)	–	–	–	–	–	–	0.82 (0.52–1.29)	0.389

*Model 1* unadjusted, *Model 2* adjusted for age, sex, current drinker, current smoker, use of antihypertensive medications, *Model 3* adjusted for age, sex, current drinker, current smoker, use of antihypertensive medications, BMI, and visceral fat area, *Model 4* adjusted for age, sex, current drinker, current smoker, use of antihypertensive medications, BMI, visceral fat area, fasting FFA, and uric acid

*BMI* body mass index, *CI* confidence interval, *FFA* free fatty acids, *OR* odds ratio

## Discussion

In this study, we firstly investigated fasting myocardial glucose uptake by using ^18^FDG-PET and visceral/subcutaneous adipose tissue areas by abdominal CT in a total of 346 individuals, who were stratified based on glucose tolerance (NGT, IFG, and T2DM). Our results demonstrated that fasting myocardial glucose uptake was markedly decreased in patients with T2DM compared to the other two groups. Reduced myocardial glucose uptake was related with a greater visceral fat area, higher concentration of circulating FFA, and uric acids, which could be related to systemic insulin resistance. Furthermore, the alteration of myocardial glucose uptake was strongly associated with T2DM, in along with visceral adiposity.

Previously, hyperglycemia, hyperinsulinemia, and disturbances of carbohydrates, fatty acids, and protein metabolism have all been correlated with prediabetes and T2DM [3, 22, 23]. Therefore, impaired glucose uptake and metabolism in not only the skeletal muscle, but also in the heart, which requires sources of energy mostly from FFA and glucose, could be correlated with insulin resistance for prediabetes and T2DM, contributing to the development of hyperglycemia [24]. The data of this study support that systemic insulin resistance is strongly related to decreased myocardial glucose uptake [25, 26]. The estimation of whole body and adipose tissue insulin resistance by HOMA-IR and Adipo-IR all showed significant negative correlations with myocardial glucose uptake in this large number of study population. Although previous studies revealed that myocardial fatty acid metabolism increased with obesity and female sex [26, 27], relationship between visceral adiposity and myocardial glucose uptake has not been studied yet. To note, this study demonstrates that greater visceral fat area, not subcutaneous is significantly associated with decreased myocardial glucose uptake as well as the presence of T2DM, even after adjustment of other confounding factors including sex and BMI. Visceral adipose tissue has proven to be causally linked to insulin resistance much greater than subcutaneous adipose tissue, by paracrine and endocrine effects from a set of cytokines, particularly high levels of tumor necrosis factor-alpha (TNF-α), low levels of adiponectin, increased macrophage accumulation, and excess of circulating FFA [28–30]. These findings are consistent with the results of the current study, showing that myocardial glucose uptake was significantly decreased, while visceral adiposity was increased with elevated levels of plasma FFA, in patients with T2DM.

In agreement with previous studies, we showed that fasting FFA was an independent predictor for myocardial glucose uptake [9, 10]. There have been conflicting findings on the relationship between the direct effect of myocardial insulin resistance on myocardial glucose uptake and the independence of increased plasma FFA [9, 25]. For example, there were reports that serum FFA concentrations suppressed by acipimox, a potent nicotinic acid derivative, affected glucose uptake in the myocardium via inhibition of lipolysis [9], and FFA levels decreased by rosiglitazone therapy were associated with the improvement in myocardial glucose uptake [31]. However, Yokoyama et al. showed that the whole body glucose disposal rate (GDR) was independently related to myocardial FDG uptake, whereas FFA was not [25]. The more prominent relation between GDR and myocardial FDG uptake than between myocardial FDG uptake and FFA in patients with diabetes suggested that insulin resistance regulates the myocardial cellular glucose FFA cycle, the so-called Randle cycle [32], and/or levels of plasma FFA. In a similar manner, Hicks et al. reported that the correlation between myocardial FDG uptake and GDR was greater than that between myocardial FDG uptake and FFA in diabetic patients [33]. In addition, the current data which visceral adiposity or uric acid as well as fasting FFA was an independent determinant for myocardial glucose uptake, also suggest that not only direct effect of elevated fasting FFA concentration but also insulin resistance may connect with myocardial metabolism.

Previous studies showed that in the normal heart under fasting conditions, FDG uptake showed variable myocardial glucose uptake because FFA is a primary source of energy, whereas glucose utilization is relatively low for the myocardial oxidative metabolism compared to glucose-loading conditions [34, 35]. However, in the T2DM heart, regulation of glucose metabolism differed from the normal heart, therefore prior studies showing that myocardial FDG uptake was significantly decreased in diabetic patients compared to normal subjects are consistent with our results [8, 34]. The underlying mechanism behind association with systemic insulin resistance and myocardial glucose metabolism has been still investigated. Cardiac myocytes utilize glucose mostly via insulin-sensitive glucose transporters (GLUT4) that are responsible for more than 50 % of all glucose uptakes in the body [36], and reduced expression and mutations of GLUT4 have been associated with diabetes [37, 38]. Recent study showed that increased insulin receptor substrate 1 (IRS1)-phosphoinositide 3-kinase (PI3K) activity with a concurrent activation of the insulin receptor was occurred with a diminished translocation of GLUT4 to the sarcolemmal membrane in the heart even in fasting status of diabetes. Also, the increase in expression of GLUT4 trafficking and docking components turned out to be dysfunction of GLUT4 vesicles in diabetic heart [39]. Therefore, whole body insulin resistance maybe connected with myocardial insulin resistance, in the condition of down-regulated sarcolemmal GLUT4, thus resulting in decreased fasting myocardial glucose uptake in this current study.

In this study, patients with T2DM had relatively low levels of HbA1c (6.8 %) and a low proportion of antidiabetic drug users (41.0 %), and most of them were newly diagnosed or well controlled diabetic patients. However, we found that myocardial glucose uptake showed a marked gradual decrease in patients with insulin resistant prediabetes and even well controlled T2DM. The relationship between hyperglycemia and development of ischemic heart disease has been well known [40, 41], but the effects of diabetes on myocardial metabolism still remain uncertain [42, 43]. Several studies have reported that a chronic shift of myocardial substrate preference in the diabetic heart resulted in a prominent decrease in glucose and lactic acid oxidation, and an increase in fatty acid oxidation [4, 5]. The effects of diabetes on myocardial metabolism are very complex, including systemic metabolic disturbances of hyperglycemia, increased FFA, down-regulation of glucose transporters, increased insufficient energy utilization of fatty acid oxidation, lipid accumulation, and lipid toxicity in cardiomyocytes [44, 45]. Therefore, the current study results could emphasize the association of diabetes and myocardial metabolism in connection with insulin resistance, and suggest that beneficial effects of an adequate glycemic control on myocardial metabolic disturbances in diabetes. These metabolic disturbances may lead to diabetic cardiomyopathy, however, further studies of the relationship between myocardial glucose uptake and cardiac function will be needed to determine the exact mechanisms [46–48].

This study had several distinguishable strengths. To our knowledge, it was the first study to investigate myocardial glucose uptake using ^18^FDG-PET in the largest study population together with the subcutaneous and visceral adiposity by abdominal CT, a gold standard method for quantification, and determinations of other metabolic parameters like HOMA-IR and Adipose-IR, which evaluated whole body insulin sensitivity, according to the glycemic status. Second, because the induction of myocardial ischemia could affect myocardial metabolism, we excluded patients not only having history of known coronary artery disease and heart failure but also having CACS over 400, who could have subclinical atherosclerosis [11]. Also, striking focal FDG uptake in the left ventricle was investigated to exclude ischemic change on PET image analysis [17]. Therefore, as the incidence of coronary artery disease in patients with diabetes is higher compared to normal healthy people, asymptomatic subjects with coronary artery disease could be excluded in this current study [49]. Finally, on PET image analysis, myocardial glucose uptake was estimated as ratio of maximum value of SUV in the myocardium and mean value of SUV in the liver. In FDG-PET for malignancy, the liver has been used as an internal standard for grading FDG uptake of whole body lesions because SUV in liver but not in other tissues stays stable over time even in patients with diffuse fatty liver disease when measured as a mean in the right lobe of the liver [19, 20, 50]. Also, the mean SUV of liver in the early images after injection (50–70 min) as same as the present study shows no dependency on blood glucose level [51]. In this current study, we obtained three mean SUVs of the right lobe of the liver, and used the values as comparators for increased FDG uptake in the heart. Therefore, the value of myocardial glucose uptake as ratio of maximum value of SUV in the myocardium and mean value of SUV in the liver is relatively consistent and reliable in this study.

The current study also had several limitations. The cross-sectional study design was insufficient to determine a causal relationship in the development of impaired myocardial glucose uptake. In addition, we did not assess cardiac function in our participants; therefore, possible correlations between myocardial glucose uptake and cardiac function would be an important topic for future studies.

## Conclusion

In conclusion, we confirmed that myocardial glucose uptake decreased in patients with T2DM, and demonstrated that there were associations between alterations of myocardial glucose uptake and increased levels of free fatty acids, uric acid, and visceral adiposity, in terms of whole body insulin resistance. Importantly, alterations of myocardial glucose uptake were related with T2DM as well as visceral adiposity. Additional studies will be needed to confirm the relationships of alterations of myocardial glucose metabolism in T2DM and the development of abnormal cardiac function.

## Abbreviations

Adipo-IR: index of adipose tissue insulin resistance
BMI: body mass index
CACS: coronary artery calcium score
CI: confidence interval
CT: computed tomography
DBP: diastolic blood pressure
DPP4: dipeptidyl peptidase-4
^18^FDG-PET: [^18^F]-fluorodeoxyglucose-positron emission tomography
FFA: free fatty acids
GDR: glucose disposal rate
HOMA-IR: homeostasis model assessment of insulin resistance
HDL: high density lipoprotein
IFG: impaired fasting glucose
IRS1: insulin receptor substrate 1
LDL: low density lipoprotein
NA: not applicable
NGT: normal glucose tolerance
OR: odds ratio
PI3K: phosphoinositide 3-kinase
SBP: systolic blood pressure
SUV: standardized uptake value
T2DM: type 2 diabetes
TG: triglycerides
TZD: thiazolidinedione
